# Polyadenylated tail length variation pattern in ultra-rapid vitrified bovine oocytes

**DOI:** 10.14202/vetworld.2016.1070-1074

**Published:** 2016-10-14

**Authors:** D. J. Dutta, Himangshu Raj, and Hiramoni Dev

**Affiliations:** Department of Veterinary Physiology, Faculty of Veterinary Science, Assam Agricultural University, Khanapara, Guwahati - 781 022, Assam, India

**Keywords:** ligation-mediated poly(A) test, oocyte, poly-adenylated tail, vitrification

## Abstract

**Aim:**

Thecurrent study aims at investigating the polyadenylated (poly[A]) tail length of morphologically high and low competent oocytes at different developmental stages. Furthermore, effect of ultra-rapid vitrification on the poly(A) tail length was studied.

**Materials and Methods:**

Fresh bovine cumulus oocyte complexes from abattoir originated ovaries were graded based on morphological characters and matured *in vitro*. Cryopreservation was done by ultra-rapid vitrification method. mRNA was isolated from different categories of oocyte and subjected to ligation-mediated poly(A) test followed by polymerase chain reaction for determining the poly(A) tail length of β actin, gap junction protein alpha 1 (GJA1), poly(A) polymerase alpha (PAPOLA), and heat shock 70 kDa protein (HSP70) transcripts.

**Results:**

GJA1, PAPOLA, and HSP70 showed significantly higher poly(A) in immature oocytes of higher competence irrespective of vitrification effects as compared to mature oocytes of higher competence.

**Conclusion:**

mRNA poly(A) tail size increases in developmentally high competent immature bovine oocytes. There was limited effect of ultra-rapid vitrification of bovine oocytes on poly(A).

## Introduction

The polyadenylated tail (poly[A] tail) on nearly all eukaryotic mRNAs plays a number of important roles in mRNA metabolism including regulating translation, mRNA stability, and transport from the nucleus. Thus, poly(A) plays a key regulatory step in gene expression and is known to be important for the developmental competence of oocyte and early embryonic development [1,2]. Posttranscriptional processes begin even while the RNA is still being transcribed and occur in a complex and highly coordinated manner, involving large complexes of proteins [3]. Poly(A) tail length regulation has been shown to play a key role in some biological processes, such as oocyte maturation, mitotic cell cycle progression [4-6]. At synthesis, the length of the poly(A) tail is generally uniform in any given system, with the absolute length being species dependent. However, in the cytoplasm, the steady state length distribution of poly(A) tail can vary dramatically for transcripts of different functional classes due to the differential, transcript-specific deadenylation rates, a disconnect between deadenylation, and decapping and/or stabilization during translation [7-9]. Thus, identifying changes in poly(A) tail length can yield insights into mRNA regulation and subsequent physiological impact.

In this study, the poly(A) tail length of β actin (ACTB), gap junction protein alpha 1 (GJA1), poly(A) polymerase alpha (PAPOLA), and heat shock 70 kDa protein (HSP70) transcripts were examined. Changes of poly(A) were examined and compared between high and low competent immature, i.e., germinal vesicle (GV) and *in vitro* matured oocytes with or without vitrification. There are several well-characterized methods for measuring poly(A)-tail length. We have made extensive use of the ligation-mediated poly(A) test (LM-PAT) assay developed by the Strickland Laboratory [10-12].

## Materials and Methods

### Ethical approval

Ethical approval was not necessary as all the ovaries were collected from government approved slaughter house.

### Oocyte recovery

Indigenous cattle ovaries were collected from government approved slaughter house and within 1½-2 h processed as per routine standard protocol. Oocytes were aspirated from 3 to 8 mm ovarian follicles with medium containing tissue culture medium (TCM-199) and supplemented with 200 mM L-glutamine solution, 0.4% bovine serum albumin, and antibiotics. A total of 1278 oocytes were subjected to this study following categorization. Cumulus oocyte complexes (COCs) were graded as follows: With an evenly granulated cytoplasm and four or more layers of cumulus cells attached were considered as morphologically high developmental competence (Group H) and oocytes with 1-3 layers of cumulus cells were considered as morphologically low developmental competence groups (Group L). Both groups (H and L) were subjected to vitrification either at the GV stage (Group G) or at the *in vitro* matured stage (Group M). RNA extraction and LM-PAT study were performed at high competent immature stage, vitrified high competent immature stage, high competent matured stage, vitrified high competent matured stage, low competent immature stage, vitrified low competent immature stage, low competent matured stage, and vitrified low competent matured stage.

Homogeneous and compact COCs were washed 4 times in holding media (Modified TCM-199, 200 mM L-glutamine solution, 10% fetal bovine serum [FBS], 0.8 M sodium pyruvate, and 50 µg/ml gentamicin and 50 µM cysteamine) by gentle pipetting and were subjected to cryopreservation by vitrification.

### *In vitro* maturation

The fresh or post-thaw vitrified normal oocytes were matured in Modified TCM-199, 200 mM L-glutamine solution, 10% FBS, 0.8 M sodium pyruvate, and 50 µg/ml gentamicin and 50 µM cysteamine supplemented with porcine-follicular stimulating hormone (5 µg/ml), 10% v/v follicular fluid, 1 µg/ml 17-β estradiol at 38.5°C in a humidified atmosphere of 5% CO_2_ for 24 h. For confirmation of maturation after 24 h, the oocytes were evaluated for morphological change and *in vitro* maturation performance under stereo zoom microscope. The oocytes with an intact zona pellucida, plasma membrane, and homogeneous cytoplasm were considered as morphologically normal in the study. *In vitro* maturation performance was assessed on the basis of expansion of cumulus cells surrounding the homogeneous oocytes [13].

### Ultra-rapid vitrification

The vitrification solutions (VS) were prepared in media consisting of TCM-199 with 10% FBS. VS I consisted of 7.5% ethylene glycol (EG) + 7.5% dimethyl sulfoxide (DMSO) and VS II consisted of 15% EG + 15% DMSO + 0.6 M sucrose. The immature bovine oocytes with cumulus cells were exposed to VS I for equilibration up to 3 min followed by 25-30 s in VS II at room temperature (22-25°C). The oocytes in VS II were immediately loaded to an open pulled straw (OPS) preloaded with 0.6 M sucrose in holding medium with air gap in between and plunged into liquid nitrogen. The OPS straws are standard 0.25 ml straw with one extremity pulled and thinned by heating. This increases the superficies/volume rate and hastens the cooling rate of small (2 µl) drop in which the oocytes is contained. The straws were stored for 7 days and then thawed in 37°C water bath for 30 s. After immersion in the water bath, oocytes were gradually rehydrated in sucrose solution. The oocytes were kept into the medium containing 0.6 M of sucrose in basic solution for 2 min. Then, they were transferred successively into the holding medium in stepwise dilution containing 0.3 M and 0.15 M of sucrose for 1 min in each. Following rehydration, oocytes were washed 3 times in holding medium. The morphological integrity of post-thaw vitrified oocytes was assessed under inverted phase contrast microscope. Oocytes having fragmented zona pellucida and absent cytoplasmic contents were not considered. The remaining morphologically normal post-thaw oocytes were taken for *in vitro* maturation.

### Isolation of mRNA from oocytes

mRNA was extracted from approximately 25 oocytes of each experimental group using Oligotex direct mRNA minikit (Qiagen, 72022). RNA quality and quantity was assessed by NanoDrop spectrophotometer.

### LM-PAT

LM-PAT is a modification of rapid amplification of cDNA ends poly(A) test designed to be more sensitive to changes in poly(A) tail length by specifically targeting the oligo(dT) anchor to the 3’ end of the poly(A) tail. To accomplish this, the poly(A) tail has been saturated with phosphorylated oligo(dT)_12–18_ [p(dT)_12–18_] at 42°C in the presence of T4 DNA ligase. The ligase creates an *in situ* oligo (dT) copy of the poly(A) tail. The determination of the poly(A) tail length was performed as described by Salles and Strickland [10] with some minor modifications.

Briefly, 1 µl of p(dT)_12–18_ (500 µg/ml) was added to 5 µl of poly A^+^ mRNA sample and heat denatured at 65°C for 5 min and transferred the tube to 42°C water bath. 17 µl of the prewarmed (42°C) mixture (4 µl ×5 first-strand buffer, 1 µl RNase inhibitor (40 U/µl), 2 µl 0.1 M dithiothreitol, 1 µl 10 mM deoxyribonucleoside triphosphate mix, 1 µl 10 mM adenosine triphosphate, 3 µl diethyl pyrocarbonate water, 1 µl T4 DNA ligase (5 U/µl), and 4 µl ×5 DNA ligase reaction buffer) was added and incubated at 42°C for 30 min. While at 42°C, 1 µl of oligo(dT) anchor was added and incubate at 23°C for 1 h then transferred to 42°C for 2 min. 2 µl of SuperScript II reverse transcriptase was added, vortex and incubated at 42°C for 1 h. Heat inactivated reverse transcriptase and ligase at 70°C for 15 min. The samples were then subjected to polymerase chain reaction (PCR) amplification and analysis.

### PCR reaction

The PCR poly(A) test requires the use of primers located close to the 3’end to provide the best PCR product size resolution [10]. The sequence of the primers and the accession number of the gene sequence used for their design are specified in Table-1. For a 25 µl reaction, 12.5 µl One Taq Hot Start ×2 master mix with standard buffer and added 0.5 µl 10 µM forward primer, 0.5 µl 10 µM anchored primer, 6.5 µl nuclease free water, and 5.0 µl template. PCR was carried out in an automated thermal cycler at 94°C for 30 s, 35 cycles of 63°C for 60 s, 68°C for 60 s, and 68°C for 5 min. The PCR product was visualized in 2% agarose gel electrophoresis for confirmation of each fragment. mRNA poly(A) levels of ACTB, GJA1, PAPOLA, and HSP70 genes in different groups of oocytes were examined. The poly(A) tail lengths of the gene of interest are the sizes of poly(A) PCR-amplified products minus the calculated length of the gene specific forward primer to the putative poly(A) start site. Poly(A) tail length assay for each transcripts was done in triplicates. Due to variation in tail size mean was calculated. Statistical analysis was performed using software SPSS version 15. Two-way ANOVA followed by *post-hoc* least significance difference was performed to compare the significance level in each oocyte category.

**Table-1:** Gene specific forward primers and anchored primer used in LM-PAT.

Genes name	Accession number	Primer (5′-3′)
ACTB	NM_173979.3	GCTGCGTTACACCCTTTTTC
GJA1	NM_174068.2	TGCAAGAGAGGTTGAAAGAGGT
PAPOLA	X63436.1	TAGGCCAGCCACATTAATCTCTA
HSP70	NM203322.2	GAAGAAGGTGCTGGACAAGTG
Anchored primer	GCGAGCTCCGCGGCCGCGT_12_	

ACTB=β actin, GJA1=Gap junction protein alpha-1, PAPOLA=Poly (A) polymerase alpha, HSP70=Heat shock 70 kDa protein, LM-PAT=Ligation-mediated poly (A) test, Poly (A)=Polyadenylated

## Results and Discussion

**Figure-1:**
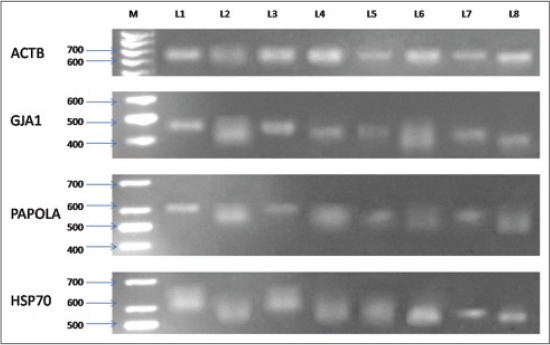
M=100 bp marker, L1=High competent immature (HG) stage, L2=High competent mature (HM) stage, L3=Vitrified high competent immature (VHG) stage, L4=Vitrified high competent mature (VHM) stage, L5=Low competent immature (LG) stage, L6=Low competent mature (LM) stage, L7=Vitrified low competent immature (VLG) stage, L8=Vitrified low competent mature (VLM) stage.

The poly(A) tail length of different transcripts (Figure-1) isolated from COCs of different developmental stage and competence with or without vitrification is presented in Table-2. Poly(A) tail length size of ACTB mRNA molecules did not show any significant change irrespective of stage of development and effect of vitrification. Whereas, GJA1, PAPOLA, and HSP70 showed significantly higher length size among immature oocytes of higher competence irrespective of vitrification effects as compared to mature oocytes of higher competence. A similar pattern has been observed among oocytes of lower competence but with decreased poly(A) tail length size.

**Table-2:** Poly (A) tail length size of different transcripts in various oocytes developmental stages.

Group	Treatment	Stage	ACTB	GJA1	PAPOLA	HSP70
Morphologically high competent oocytes	Fresh	Immature	168±1.85^a^	146±2.40^a^	132±3.71^a^	116±2.08^a^
		Mature	162±1.20^a^	102±4.63^c^	89±2.08^b^	70±1.76^b^
	Vitrified	Immature	165±1.67^a^	139±0.33^a^	128±1.85^a^	111±1.45^a^
		Mature	159±2.03^a^	102±11.05^c^	86±1.52^b^	67±3.60^b^
Morphologically low competent oocytes	Fresh	Immature	164±2.90^a^	111±8.62^b^	73±7.94^c^	67±6.24^c^
		Mature	161±4.58^a^	77±4.72^d^	31±6.33^d^	33±7.75^d^
	Vitrified	Immature	162±2.33^a^	105±3.53^c^	69±9.53^c^	60±2.64^c^
		Mature	158±6.88^a^	77±6.43^d^	31±2.40^d^	31±7.00^d^

Different superscript in a column differ significantly (p<0.05). ACTB=β actin, GJA1=Gap junction protein alpha-1, PAPOLA=Poly (A) polymerase alpha, HSP70=Heat shock 70 kDa protein, Poly (A)=Polyadenylated

The results obtained in this study support earlier observation of poly(A) tail differences in mRNA from bovine oocytes at GV stage and metaphase II stage [14]. The poly(A) level of ACTB transcript remain unchanged during the initial phase of development and are not related to the different level of developmental competence, an indicative of nonhousekeeping gene. Bilodean-Goeseels and Schultz [15] and Brevini-Gandolfi *et al*. [16] indicated ACTB as housekeeping gene and their translation efficiency, presumably, remains unchanged. Classical housekeeping genes are ubiquitously expressed and involved in protein biosynthesis and energy metabolism [17]. Developmental competence is acquired by completion of oocyte maturation. Given that maturing oocytes are transcriptionally quiescent (as are early embryos), they depend on posttranscriptional regulation of stored transcripts for protein synthesis, which is largely mediated by translational repression and deadenylation of transcripts within the cytoplasm [2]. Whereas GJA1, PAPOLA, and HSP70 follow the pattern of progressive deadenylation during oocytes maturation that showed more poly(A) among high competent oocytes. Increase in poly(A) tail length generally correlate with increase option for translation and decreases correlate with repression [18]. Poor/low developmental competence is associated with defective (generally lower) poly(A) level of the maternal transcript [16].

Embryonic development is supported by maternal mRNA and protein synthesized and stored during oogenesis. The stored maternal information is also involved in development after the so-called maternal-embryonic transition since it has been shown in mice that protein synthesized from maternal mRNA are very stable throughout preimplantation development [19]. The extent of poly(A) tail at the 3’end of mRNA transcript has emerged as an important regulatory element from determining their stability. The observation in the present experiment supports the hypothesis that morphologically competence COCs and control of poly(A) represents a key regulatory step in gene expression and early embryonic development in bovine. The observation recorded in the present experiment indicated a link that a morphologically lower COCs has reduced embryonic developmental competence and an altered pattern of maternal transcripts poly(A). There was limited effect of ultra-rapid vitrification of bovine COCs in respect to altered pattern of poly(A) in this study. Succu *et al*. [20] reported lower oocyte transcripts (mRNA) abundance following vitrification. However, Monzo *et al*. [21] stated that slowly frozen and vitrified metaphase oocytes displayed specific gene expression signatures. Survival and fertilization rates were higher when vitrified rather than slowly frozen human mature oocytes [22].

The difference of poly(A) pattern that exist in COCs of different developmental stage and competence were not eliminated by the effect of vitrification using ultra-rapid freezing with OPS. In general, poly(A) is associated with translational activation, whereas deadenylation is associated with translational silencing [23].

## Conclusion

The poly(A) tail length of GJA1, PAPOLA, and HSP70 mRNA molecules showed significantly higher length size among immature oocytes of higher competence irrespective of vitrification effects as compared to mature oocytes of higher competence. A similar pattern has been observed among oocytes of lower competence but with decreased poly(A) tail length size. Morphologically poor COCs have reduced embryonic developmental competence and an altered pattern of maternal transcripts poly(A). There was limited effect of vitrification of bovine COCs in respect to altered pattern of poly(A).

## Authors’ Contributions

DJD and HR were involved in the design of this research work. The research was done by DJD, HR, and HD. DJD has monitored all the activities being a supervisor. HD and HR drafted and revised the manuscript. All authors read and approved the final manuscript.
